# Association Between Haemoglobin Variant Phenotypes and Red Blood Cell Parameters Among Pregnant Women: A Hospital‐Based Cross‐Sectional Study in Ghana

**DOI:** 10.1155/bmri/9416451

**Published:** 2026-09-10

**Authors:** Gabriel Abbam, Bertrand Nii Martey Nmeterson, Samuel Kwasi Appiah, Charles Nkansah, Moses Banyeh, Samira Daud, Muniru Mohammed Tanko, Felix Osei-Boakye, Sophia Owusua Amankwah, Simon Bannison Bani, Ejike Felix Chukwurah

**Affiliations:** ^1^ Department of Haematology, School of Allied Health Sciences, University for Development Studies, Tamale, Ghana, uds.edu.gh; ^2^ Department of Medical Laboratory Sciences, Faculty of Health Science and Technology, Ebonyi State University, Abakaliki, Nigeria, ebsu-edu.net; ^3^ Department of Biomedical Laboratory Sciences, School of Allied Health Sciences, University for Development Studies, Tamale, Ghana, uds.edu.gh; ^4^ Laboratory Department, Ga North Municipal Hospital, Accra, Greater Accra, Ghana; ^5^ Department of Immunology and Immunodiagnostics, School of Allied Health Sciences, University for Development Studies, Tamale, Ghana, uds.edu.gh; ^6^ Department of Medical Laboratory Technology, Faculty of Applied Science and Technology, Sunyani Technical University, Sunyani, Ghana, stu.edu.gh; ^7^ Laboratory Department, Ga West Municipal Hospital, Accra, Greater Accra, Ghana

## Abstract

Haemoglobin (Hb) variants arising from mutations in globin chains can alter red blood cell (RBC) parameters. This may complicate the interpretation of pregnancy‐related haematological changes, particularly in sub‐Saharan Africa, where both anaemia in pregnancy and Hb variants are highly prevalent. The association between these variants and red cell parameters among Ghanaian pregnant women remains insufficiently characterised. This cross‐sectional study investigated the association between Hb variant phenotypes and red cell parameters, and assessed whether such variants were independently associated with anaemia among 400 pregnant women at the Ga West Municipal Hospital, Greater Accra Region, Ghana. Full blood counts and Hb phenotyping by cellulose acetate electrophoresis at pH 8.4 were performed. Multivariable linear and Firth′s penalised‐likelihood logistic regression models, adjusted for maternal age, body mass index, gestational trimester, parity and haematinic supplementation, were used to estimate associations. Phenotype distribution was HbA 73.2%, HbAS/HbAC 25.3% and HbS/HbC/HbSC 1.5%; anaemia prevalence was 87.5%. Compared with HbA, HbAS/HbAC carriers had lower haematocrit (adjusted *β* = –1.14%, *p* = 0.036), mean cell volume (adjusted *β* = –4.05 fL, *p* < 0.001), mean cell Hb (adjusted *β* = –0.89 pg, *p* = 0.005) and red cell distribution width‐standard deviation (RDW‐SD) (adjusted *β* = –2.79 fL, *p* < 0.001); HbS/HbC/HbSC reductions were larger for haematocrit (adjusted *β* = –6.29%, *p* = 0.005) and mean cell volume (adjusted *β* = –6.87 fL, *p* = 0.001). Hb phenotype was not independently associated with anaemia, but the adjusted odds of anaemia in the second trimester were significantly higher than in the first (adjusted odds ratio = 3.45, *p* = 0.001). Hb variant phenotypes were associated with a microcytic, hypochromic red cell profile, whereas gestational trimester, rather than phenotype, was the dominant correlate of anaemia. Given the single‐centre, cross‐sectional design and the small number of pregnant women with homozygous or compound heterozygous phenotypes, these findings are hypothesis‐generating. They support phenotype‐ and trimester‐aware interpretation of red cell parameters in antenatal care; however, whether such interpretation improves maternal outcomes requires evaluation in larger, multicentre studies.

## 1. Introduction

Human embryonic, foetal and adult developmental stages have distinct oxygen requirements that are met by specific structural haemoglobins (Hbs), each of which is a tetramer of two pairs of unlike globin chains with a haem group per chain [1–3]. The foetal stage is mainly characterised by the presence of foetal haemoglobin (HbF) composed of alpha and gamma (α2*γ*2) globin chains, whereas normal adult Hb consists of a major haemoglobin A (HbA) and a minor HbA_2_ component made of alpha‐beta (α2*β*2) and alpha‐delta (α2*δ*2) globin chains, respectively [1, 3]. Structural changes due to mutations in globin chains produce Hb variants with altered morphology, stability or physiological properties (oxygen affinity), which may result in clinical disorders [4]. Globally, several structural Hb variants have been identified; however, haemoglobin S (HbS), the most pathological variant responsible for sickle cell disease, together with haemoglobins C, D and E (HbC, HbD and HbE), are the most common [1, 5]. HbS is predominantly seen in Africa, particularly sub‐Saharan Africa, with HbC restricted to some parts of North and West Africa [6–8]. HbD and HbE are predominant in the Middle East, India and Southeast Asia [7]. The frequency distribution of these Hb variants has immense public health implications, especially in regions where these mutations are endemic [7, 9].

The structural and functional alterations in these variant Hbs subsequently affect the peripheral red blood cell (RBC) count, Hb concentration and indices such as haematocrit (Hct), mean cell volume (MCV), mean cell haemoglobin (MCH), mean cell haemoglobin concentration (MCHC) and red cell distribution width (RDW), which are key parameters used in a full blood count (FBC) to diagnose and monitor blood dyscrasias [6, 8, 10]. These red cell parameters and indices are also indispensable in monitoring and managing pregnancy, serving as key indicators of maternal and foetal health because of the enormous physiological changes characteristic of pregnancy [11, 12]. A haemato‐physiological condition usually observed during pregnancy is anaemia, which, when severe, adversely affects birth outcomes because of impaired or reduced oxygen delivery to both maternal tissues and the foetus [13]. In anaemia, there is a reduction in Hb concentration or the number of RBCs, leading to a decrease in the oxygen‐carrying capacity of the blood [14]. Early detection and monitoring of anaemia through the assessment of red cell parameters are essential for preventing complications and ensuring favourable maternal and foetal health [12].

Public health strategies for the management of pregnancy‐related anaemia in regions where variant Hbs are endemic include nutritional assessment and iron studies, as well as Hb‐type screening for hereditary conditions such as sickle cell disease and thalassaemia [12, 15, 16]. In Ghana, although these strategies are implemented during antenatal care (ANC), little attention is paid to FBC parameters, especially red cell indices, in relation to the anaemia status of pregnant women with abnormal Hb variants other than HbS [17, 18]. However, these “overlooked” abnormal Hb variants have the potential to influence red cell indices, leading to atypical haematological results which can be misinterpreted as normal pregnancy‐related changes and subsequently increase the risk of maternal and foetal complications [6]. Two specific gaps therefore remain. First, antenatal studies from Ghana and the wider sub‐region have largely reported the frequency distribution of Hb variant phenotypes [19–23] without quantifying how each phenotype group relates to individual red cell parameters. Second, few analyses adjusted for maternal and obstetric factors, particularly the gestational trimester, which independently shapes red cell parameters in pregnancy [24–28]; hence, it remains unclear whether the reported differences reflect the Hb phenotype itself or physiological haemodilution during pregnancy. To address these gaps, this study investigated the relationship between Hb variant phenotypes and RBC parameters, including anaemia status, among pregnant women at a municipal hospital in the Greater Accra Region of Ghana. By adjusting for maternal age, body mass index (BMI), gestational trimester, parity and haematinic supplementation, this study sought to distinguish the effects of the inherited phenotype from that of the gestational stage on the interpretation of antenatal red cell parameters.

## 2. Methods

### 2.1. Ethics Approval and Consent to Participate

Ethical approval for this study was obtained from the Institutional Review Board of the University for Development Studies, Tamale (UDS/RB/0159/25). Permission was also sought from the management of the Ga West Municipal Hospital (GWMH) before the commencement of the study. The purpose of the study was explained to each participant. Written and verbal informed consent was obtained from all participants.

### 2.2. Study Design and Setting

This cross‐sectional study was conducted between June and September 2025. The study involved pregnant women who attended the ANC clinic at the GWMH (lat 5°42 ^′^N; long 0°18 ^′^W) in Amasaman (lat 5.71°N; long 0.3°W), the administrative capital of the Ga West Municipality (lat 5°39 ^′^N–5°48 ^′^N; long 0°12 ^′^W–0°22 ^′^W) in the Greater Accra Region of Ghana. The municipality is among the 29 Metropolitan, Municipal and District Assemblies (MMDAs) that make up the region and serves as the western entry corridor to the national capital. The municipality has a resident population of 314,299 and comprises heterogeneous settlement patterns characterised by a mix of urban, peri‐urban and rural communities [29, 30]. The GWMH is a government‐owned 105‐bed capacity primary referral hospital strategically located in the municipality to provide comprehensive healthcare services, including outpatient care, inpatient management, obstetric care, emergency services and laboratory diagnostics to the rapidly growing and ethnically diverse population of the municipality and its surroundings [31, 32]. The ANC clinic serves pregnant women from across the municipality and the neighbouring areas. In addition, the laboratory infrastructure of the hospital supports routine haematological investigations, including FBC and Hb variant screening, which are essential components of standard ANC protocols in Ghana.

### 2.3. Study Population, Eligibility Criteria and Sample Size

Pregnant women who attended the ANC clinic at the GWMH during the study period were eligible for inclusion. The inclusion criteria were a documented confirmation of pregnancy in the participant′s ANC record (by urine pregnosticon test and ultrasound scan performed at the first ANC visit) and completion of the informed consent form. Pregnant women were excluded if they had a known history of chronic illness (including HIV/AIDS, hepatitis B infection, diabetes, hypertension and renal or hepatic dysfunction), adverse obstetric complications with haematological implications (such as antepartum haemorrhage or pre‐eclampsia), blood transfusion within 3 months prior to enrolment, malaria or severe illness at the time of recruitment. Because red cell parameters in sickle cell disease vary significantly between crisis and steady state, pregnant women subsequently found to have homozygous or compound heterozygous phenotypes were required to have been in a clinically steady state at the time of sampling. Steady state was defined a priori as the absence of any vaso‐occlusive or painful crisis in the 30 days preceding sample collection, the absence of acute illness or hospital admission at recruitment and no blood transfusion in the preceding 3 months, as ascertained by interviewer questioning and cross‐verified against ANC records. All six pregnant women with the HbS/HbC/HbSC phenotype met these criteria. To determine the required sample size, a reported anaemia prevalence of 42.4% among pregnant women attending ANC in Ghana [33] was used. Applying the Cochran [34] formula for single‐proportion cross‐sectional studies at a 95% confidence level and 5% margin of error yielded a minimum sample size of 376 participants. To accommodate an anticipated 10% postrecruitment exclusion rate, 417 pregnant women were consecutively recruited at the ANC clinic during the study period. Following interviewer‐administered eligibility screening, 17 women were excluded on the basis of the predefined exclusion criteria (a documented history of hypertension, diabetes mellitus, hepatitis B infection or HIV infections, established by interviewer‐administered questioning and cross‐verified against the participants′ current ANC records), yielding a final analytic sample of 400 participants. All 400 included participants were additionally confirmed to be negative for intestinal flagellates and helminths on stool examination, and for malaria parasites on blood films, prior to subsequent analyses.

### 2.4. Bias

To minimise selection bias, eligible pregnant women were enrolled consecutively at the ANC clinic during the study period using uniformly applied inclusion and exclusion criteria. To limit information bias, all interviewer‐administered questionnaires were completed by trained research assistants using a standardised instrument administered in the participant′s preferred language, and all laboratory analyses were performed at a single hospital laboratory using daily‐calibrated equipment and quality‐controlled reagents. Confounding was addressed through multivariable adjustment for a priori selected demographic, obstetric and clinical covariates as described below.

### 2.5. Outcomes

The primary outcome was the red cell parameters (Hct, MCV, MCH and RDW‐SD) analysed as continuous variables across the Hb phenotype groups. RBC count, Hb concentration and MCHC were examined in an exploratory comparison across the six individual phenotypes and were not carried forward into the regression models because they did not differ significantly between phenotypes (Table S3). The secondary outcome was anaemia in pregnancy, analysed dichotomously and, among anaemic pregnant women, by World Health Organisation severity grade. The distribution of Hb variant phenotypes in this antenatal population was reported as a descriptive outcome.

### 2.6. Anaemia Definition

In this study, Hb levels were classified according to the World Health Organisation′s definition for pregnant women, with anaemia defined as Hb < 11.0 g/dL and further graded as mild (10.0–10.9 g/dL), moderate (7.0–9.9 g/dL) or severe (< 7.0 g/dL) [35].

### 2.7. Data/Sample Collection and Analysis

Socio‐demographic data (age, level of education, marital status and religion) and obstetric data (gestational age, parity, blood pressure, BMI and haematinic supplementation status) were collected using an interviewer‐administered questionnaire designed specifically for this study and administered in English, Twi, Ga and Ewe by trained research assistants at the time of recruitment. Interview responses related to known medical history (including hypertension, diabetes, hepatitis B infection and HIV infection) were cross‐verified against the participants′ current ANC records to confirm eligibility. Medication use during pregnancy was assessed in two ways. The current use of haematinic supplements (iron and/or folic acid preparations dispensed as part of routine ANC) was recorded as a binary variable and retained as a covariate in all the multivariable models. Receipt of intermittent preventive treatment in pregnancy with sulfadoxine‐pyrimethamine (IPTp‐SP) was also recorded. Specific drug names, formulations, doses, duration of use and adherence were not documented. No record of hydroxyurea or other disease‐modifying therapy for sickle cell disease was found in the ANC records of any participant, and such therapy is not routinely offered during pregnancy in this setting. These medicines were also not the subject of a specific question in this study; however, their use could not be positively excluded. Gestational age was not rederived for this study; it was abstracted from the participants′ ANC records, in which it was established at booking from the reported last menstrual period and confirmed by the ultrasound scan performed at the first antenatal visit. Participants were then classified by gestational trimester as first (0–13 completed weeks), second (14–27 weeks) or third (28–41 weeks).

Approximately 3 mL of venous blood was collected aseptically from each enrolled pregnant woman and aliquoted into two ethylenediaminetetraacetic acid (EDTA) tubes. Two millilitres (2 mL) was used for malaria testing by microscopy and FBC analysis using an automated 5‐part URIT‐5160 haematology analyser (URIT‐Medical Electronics Company Ltd., China) following the manufacturer′s instructions. One millilitre (1 mL) was used for sickling screening using the 2% sodium metabisulphite method and Hb phenotyping by cellulose acetate electrophoresis at pH 8.4, as described by Old et al. [36]. Phenotypes were assigned based on the pattern of band migration, interpreted together with the sickling test result, which distinguished S‐containing from non‐S‐containing phenotypes. No confirmatory testing was performed using high‐performance liquid chromatography (HPLC), capillary electrophoresis, citrate agar electrophoresis at an acidic pH or molecular analysis. Because HbC comigrates with HbE and HbA2 at an alkaline pH, the assignment of HbC‐containing phenotypes (HbAC, HbC and HbSC) is presumptive and interpreted accordingly. Each pregnant woman was provided with two sterile, dry, wide‐mouthed plastic collection cups (30 mL capacity) with screw lids. Each cup was labelled with a unique identification number, age and gestational term (trimester). Participants provided midstream urine and stool samples for routine urinalysis and stool examination, respectively. Stool examination was performed to screen for intestinal flagellates and helminths. Urinalysis was conducted to detect haematuria, proteinuria, glucosuria, ketonuria, and nitrituria using URIT 10V, a 10‐parameter urine reagent strip (URIT‐Medical Electronics Company Ltd., China). All laboratory analyses were performed at the GWMH.

### 2.8. Quality Control (QC)

The haematology analyser was calibrated daily using manufacturer‐supplied calibrators, and QC was performed using low‐, normal‐ and high‐level control samples. FBC analysis was performed only when all QC results were within the acceptable ranges set by the manufacturer. The 2% sodium metabisulphite solution was prepared fresh daily, and a known sickling‐positive sample and a known negative sample were used to verify the reactivity of the reagent before testing the participant samples. For Hb electrophoresis, a haemolysate Hb AFSC control (Sebia, Parc Technologique Léonard de Vinci, France) was run alongside the participant samples for Hb band identification. The URIT 10V strips were stored at the manufacturer′s recommended temperature (2°C–30°C), and each lot of reagent strips was subjected to quality control by comparison with known negative and positive controls to ensure valid test results. All wet slides for urine and stool microscopy were examined in duplicates.

### 2.9. Data Processing and Analysis

Data were entered into a Microsoft Excel spreadsheet (Microsoft Corporation, Redmond, Washington, United States) and exported to Stata Version 19.5 (StataCorp LLC, College Station, Texas, United States) for statistical analysis. Complete‐case analysis was performed and no imputation was required as all 400 included pregnant women had complete data on the primary variables of interest. Categorical variables were presented as frequencies and percentages, whereas continuous variables were summarised as mean with standard deviation (SD) for normally distributed data, and medians with interquartile ranges (IQR) for non‐normally distributed data, when normality was assessed using the Shapiro–Wilk test. Categorical variables were compared across Hb phenotype groups using Fisher′s exact test because of the small expected cell counts from the limited size of the HbS/HbC/HbSC group (*n* = 6). Continuous variables were compared using the Kruskal–Wallis test and Dunn′s post hoc pairwise analysis. Univariable and multivariable linear regression analyses were used to assess the association between Hb phenotypes and red cell parameters, whereas univariable and multivariable logistic regression were used to assess the association between Hb phenotypes and anaemia status. Because the HbS/HbC/HbSC phenotype group had no nonanaemic participants (a situation known as complete separation), Firth′s penalised‐likelihood logistic regression was used to obtain stable coefficient estimates [37]. For both regression analyses, HbA was used as the reference category. Covariates included in the multivariable models were selected a priori based on biological plausibility and prior evidence of their association with maternal red cell parameters during pregnancy [24–28] and comprised maternal age, BMI, gestational trimester, parity and haematinic supplementation. Robust (Huber–White) standard errors were applied to the linear regression models to address potential heteroscedasticity, and multicollinearity among predictor variables was assessed using the variance inflation factor (VIF), with values below 5 considered acceptable. Linear regression results are reported as adjusted *β* coefficients with 95% confidence intervals (CIs), and logistic regression results as adjusted odds ratios (aORs) with 95% CI. Statistical significance was set at *p* < 0.05. This study was conducted and reported in accordance with the Strengthening the Reporting of Observational Studies in Epidemiology (STROBE) statement for cross‐sectional studies [38].

### 2.10. Statistical Power and Precision

The sample size of this study was calculated to estimate the prevalence of anaemia rather than to compare phenotype subgroups, and the achieved sample of 400 pregnant women estimated an anaemia prevalence of 87.5% with a 95% CI of 84.3% to 90.7% (half‐width 3.2%). To quantify the precision achieved for the analytical comparisons, the minimum detectable effects were derived from the standard errors of the fitted multivariable models at a two‐sided *α* of 0.05 and 80% power. The observed (post hoc) power was deliberately not computed because it is a one‐to‐one function of the obtained *p*‐value and therefore conveys no information beyond that already contained in the CI [39, 40]. With 293 pregnant women in the HbA reference group and 101 in the HbAS/HbAC group, the study could detect a standardised between‐group difference of 0.32 SDs with 80% power, corresponding to 1.5% for Hct, 3.0 fL for MCV, 0.88 pg for MCH and 1.8 fL for RDW‐SD. With only six pregnant women in the HbS/HbC/HbSC group, the corresponding minimum detectable difference was 1.16 SDs, equivalent to 6.3% for Hct, 5.5 fL for MCV, 3.5 pg for MCH and 4.5 fL for RDW‐SD. For the binary anaemia outcome, the study had 80% power to detect an adjusted odds ratio of 2.99 or greater (or 0.33 or smaller) in the HbAS/HbAC group, whereas the corresponding threshold in the HbS/HbC/HbSC group was 69, indicating that this comparison was uninformative. Therefore, estimates are reported with 95% CI throughout, and the precision of each estimate is considered alongside its *p*‐value when interpreting null findings.

## 3. Results

### 3.1. Socio‐Demograph, Obstetric, Clinical and Anthropometric Characteristics

**Figure 1:**
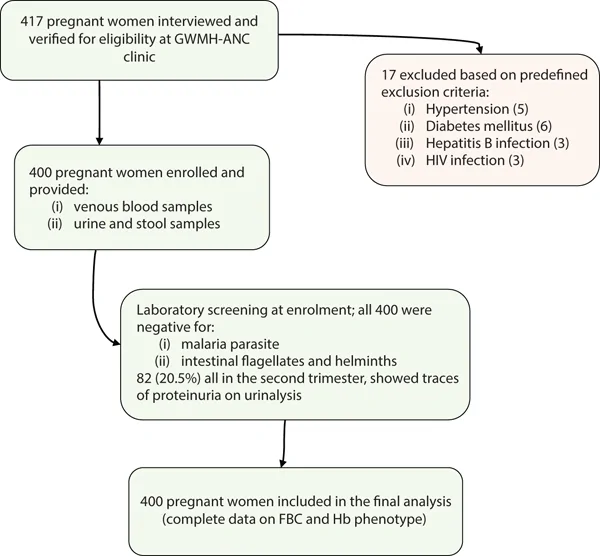
Flow of participants through the study.

A total of 417 pregnant women attending the antenatal clinic at the GWMH during the study period were screened for eligibility. Of these, 17 were excluded during the interviewer‐administered screening on the basis of the predefined exclusion criteria (a documented history of hypertension, diabetes mellitus, hepatitis B infection or HIV infection, established by interviewer‐administered questioning and cross‐verified against the participants′ current ANC records). A final analytic sample of 400 pregnant women, all with complete data, was retained for analyses (Figure 1). The 400 pregnant women enrolled in this study had a mean age of 28.5 ± 6.4 years. Notably, traces of proteinuria were detected on urinalysis in 82 women (20.5%), all of whom were in their second trimester of pregnancy Table S1). Haematinic supplementation was reported by 152 (38.0%) of the pregnant women, and 205 (51.3%) reported having received at least one dose of IPTp‐SP by the time of enrolment. Neither haematinic use (*p* = 0.631) nor IPTp‐SP receipt (*p* = 0.126) differed significantly across the Hb phenotype groups. Hb electrophoresis for variant phenotypes revealed that 293 (73.2%) pregnant women had the normal HbA phenotype, whereas 101 (25.3%) were carriers of variant Hb (heterozygous phenotypes, HbAS/HbAC), including 55 (13.8%) with the sickle cell trait (HbAS) and 46 (11.5%) with the HbAC trait. Additionally, 6 (1.5%) pregnant women were identified with homozygous or compound heterozygous phenotypes (2 each with HbS, HbC and HbSC). The socio‐demographic, obstetric, clinical and anthropometric characteristics of the participants were compared across three biologically coherent Hb phenotype groups (HbA, HbAS/HbAC and HbS/HbC/HbSC). There were no statistically significant differences in age, educational background, marital status, religion, gestational trimester, parity, haematinic supplementation, anaemia status and blood pressure of participants across the three Hb phenotype groups (all *p* > 0.05). However, the BMI differed significantly between the groups (*p* = 0.005), with the HbS/HbC/HbSC group having the lowest median BMI (23.2 kg/m^2^, [IQR] 21.3–23.5) compared with the HbA and HbAS/AC groups (Tables 1 and 2). A post hoc Dunn′s pairwise test with Bonferroni adjustment revealed that the BMI of the HbS/HbC/HbSC group was significantly lower than that of the HbA (adjusted *p* = 0.016) and HbAS/HbAC groups (adjusted *p* = 0.004). No significant difference in BMI was observed between the HbA and HbAS/HbAC groups (adjusted *p* = 0.091) (Table S2).

**Table 1:** Socio‐demographic and obstetric characteristics of participants by Hb phenotype groups.

Variable	HbA (*n* = 293)	HbAS/AC (*n* = 101)	HbS/HbC/HbSC (*n* = 6)	Total (*N* = 400)	*p* ^a^
Age groups, *n*(%)					0.833
14–21 years	38 (13.0)	15 (14.8)	2 (33.3)	55 (13.8)	
22–29 years	127 (43.3)	41 (40.6)	2 (33.3)	170 (42.5)	
30–37 years	104 (35.5)	35 (34.7)	2 (33.3)	141 (35.2)	
38–48 years	24 (8.2)	10 (9.9)	0 (0.0)	34 (8.5)	
Educational background, *n*(%)					0.355
Basic	141 (48.1)	55 (54.5)	6 (100.0)	202 (50.5)	
Secondary	103 (35.2)	29 (28.7)	0 (0.0)	132 (33.0)	
Tertiary	25 (8.5)	10 (9.9)	0 (0.0)	35 (8.7)	
None	24 (8.2)	7 (6.9)	0 (0.0)	31 (7.8)	
Marital status, *n*(%)					0.911
Married	183 (62.5)	61(60.4)	4 (66.7)	248 (62.0)	
Not married	110 (37.5)	40 (39.6)	2 (33.3)	152 (38.0)	
Religion, *n*(%)					0.195
Christian	246 (84.0)	91 (90.1)	4 (66.7)	341 (85.2)	
Muslim	46 (15.7)	10 (9.9)	2 (33.3)	58 (14.5)	
Other	1 (0.3)	0 (0.0)	0 (0.0)	1 (0.3)	
Gestational trimester^b^, *n*(%)					0.804
First	145 (49.5)	44 (43.6)	3 (50.0)	192 (48.0)	
Second	123 (42.0)	46 (45.5)	3 (50.0)	172 (43.0)	
Third	25 (8.5)	11 (10.9)	0 (0.0)	36 (9.0)	
Parity, *n*(%)					0.744
Nulliparous	94 (32.1)	40 (39.6)	3 (50.0)	137 (34.3)	
Primiparous	82 (28.0)	22 (21.8)	1 (16.7)	105 (26.2)	
Multiparous	104 (35.5)	36 (35.6)	2 (33.3)	142 (35.5)	
Grand multiparous	13 (4.4)	3 (3.0)	0 (0.0)	16 (4.0)	
Haematinics usage, *n*(%)					0.631
Yes	113 (38.6)	38 (37.6)	1 (16.7)	152 (38.0)	
No	180 (61.4)	63 (62.4)	5 (83.3)	248 (62.0)	
IPTp‐SP receipt^c^, *n*(%)					0.126
Yes	147 (50.2)	57 (56.4)	1 (16.7)	205 (51.3)	
No	146 (49.8)	44 (43.6)	5 (83.3)	195 (48.7)	
Anaemia status^d^, *n*(%)					0.085
Non‐anaemic	41 (14.0)	9 (8.9)	0 (0.0)	50 (12.5)	
Mild anaemia	74 (25.3)	29 (28.7)	0 (0.0)	103 (25.8)	
Moderate anaemia	167 (57.0)	60 (59.4)	4 (66.7)	231 (57.8)	
Severe anaemia	11 (3.8)	3 (3.0)	2 (33.3)	16 (4.0)	

*Note:* Data are presented as frequencies (percentages).

Abbreviations: %, percentage; Hb, haemoglobin; *N*, total number of participants; *n*, number in subgroup.

^a^Fisher′s exact test was used to compare categorical variables because of the small expected cell counts from the limited size of the HbS/HbC/HbSC phenotype group. Phenotype assignment was based on cellulose acetate electrophoresis at pH 8.4, read together with the 2% sodium metabisulphite sickling test. HbC‐containing phenotypes were not confirmed by HPLC or capillary electrophoresis and are therefore presumptive.

^b^Gestational trimester (gestational age) category: first trimester: 0–13 weeks; second trimester: 14–27 weeks; third trimester: 28–41 weeks.

^c^IPTp‐SP: intermittent preventive treatment in pregnancy with sulfadoxine‐pyrimethamine, receipt of at least one dose by the time of enrolment.

^d^Anaemia status: determined by classifying haemoglobin (Hb) levels as anaemic (Hb < 11.0 g/dL), with severity further graded as mild (Hb 10.0–10.9 g/dL), moderate (Hb 7.0–9.9 g/dL) and severe (Hb < 7.0 g/dL) based on the World Health Organisation′s definition for pregnant women [35].

**Table 2:** Clinical and anthropometric characteristics by Hb phenotype groups.

Variable	HbA (*n* = 293)	HbAS/AC (*n* = 101)	HbS/HbC/HbSC (*n* = 6)	Total (*N* = 400)	*p*
Systolic BP (mmHg) median (IQR)	113 (108–119)	114 (107–119)	114 (112–119)	113 (108–119)	0.738
Diastolic BP (mmHg) median (IQR)	69 (63–77)	69 (63–76)	71 (66–72)	69 (63–76)	0.991
BMI (kg/m^2^) median (IQR)	26.6(23.4–29.4)	27.8(24.4–30.1)	23.2(21.3–23.5)	26.8(23.6–29.7)	0.005

*Note:* Data are presented as median (interquartile range). Kruskal–Wallis test was used for systolic, diastolic blood pressure and body mass index comparisons.

Abbreviations: BMI, body mass index; BP, blood pressure; Hb, haemoglobin; IQR, 25^th^, 75^th^ interquartile ranges.

### 3.2. Association Between Hb Phenotypes and RBC Parameters

An initial exploratory comparison of seven red cell parameters (RBC, Hb, Hct, MCV, MCH, MCHC and RDW‐SD) across the six individual Hb phenotypes (HbA, HbAS, HbAC, HbS, HbC and HbSC) is presented in Table S3. Statistically significant differences were observed in Hct (*p* = 0.032), MCV (*p* = 0.007), MCH (*p* = 0.033) and RDW‐SD (*p* = 0.001), whereas RBC, Hb and MCHC (all *p* > 0.05) did not differ significantly between the phenotypes. Because of the very small sizes of the homozygous and compound heterozygous phenotype subgroups (HbS, HbC and HbSC), subsequent inferential analyses were performed on the four parameters showing significant between‐group differences, using three biologically coherent phenotype groups (HbA as reference, heterozygous phenotypes [HbAS/HbAC], and homozygous or compound heterozygous phenotypes [HbS/HbC/HbSC]).

In the univariable (unadjusted) linear regression analysis, both the HbAS/HbAC and HbS/HbC/HbSC phenotype groups showed lower mean values across the selected red cell parameters than the HbA reference group, with several reaching statistical significance (Table 3). Pregnant women in the HbAS/HbAC phenotype group had significantly lower Hct (*β* = –1.29%, 95% CI: −2.38, −0.20; *p* = 0.021), MCV (*β* = −4.05 fL, 95% CI: −6.10, −2.00; *p* < 0.001), MCH (*β* = −0.89 pg, 95% CI: −1.49, −0.29; *p* = 0.004) and RDW‐SD (*β* = −2.80 fL, 95% CI: −4.03, −1.57 *p* < 0.001) values than the HbA group. The HbS/HbC/HbSC phenotype group also showed a statistically significant and numerically larger reduction in Hct (*β* = −6.40%, 95% CI: −10.50, −2.29; *p* = 0.002) and MCV (*β* = −6.45 fL, 95% CI:−10.29,−2.61; *p* = 0.001); reductions in MCH and RDW‐SD in this group were in the same direction but did not reach statistical significance. After adjusting for maternal age, BMI, gestational trimester, parity and haematinic supplementation, the Hb phenotype groups remained independently associated with the selected red cell parameters (Table 4). The HbAS/HbAC phenotype group had significantly lower Hct (adjusted *β* = −1.14%, 95% CI: −2.20, −0.08; *p* = 0.036), MCV (adjusted *β* = −4.05 fL, 95% CI: −6.15, −1.95; *p* < 0.001), MCH (adjusted *β* = −0.89 pg, 95% CI: −1.51, −0.28; *p* = 0.005) and RDW‐SD (adjusted *β* = −2.79 fL, 95% CI: −4.03, −1.55; *p* < 0.001) than the HbA phenotype group. The HbS/HbC/HbSC phenotype group showed numerically larger and statistically significant reductions in Hct (adjusted *β* = −6.29%, 95% CI: −10.66, −1.92; *p* = 0.005) and MCV (adjusted *β* = −6.87 fL, 95% CI:−10.73, −3.01; *p* = 0.001) values; reductions in MCH and RDW‐SD were in the same direction but did not reach statistical significance. Among the covariates, only gestational trimester was consistently and independently associated with the red cell parameters: Hct was 1.92% lower in the second trimester (*p* < 0.001) and 3.34% lower in the third trimester (*p* < 0.001) than that in the first trimester and RDW‐SD was 2.43 fL lower in the third trimester (*p* = 0.015) than in the first trimester, and the difference in the second trimester was not statistically significant. Maternal age, BMI, parity and haematinic supplementation were not independently associated with any of the four RBC parameters examined. No evidence of multicollinearity was observed in the multivariable models (mean VIF = 1.30; all individual VIFs < 2.1).

**Table 3:** Univariable linear regression of haemoglobin phenotype groups and selected red cell parameters of pregnant women.

Red cell indices (units)	HbA mean (reference)	HbAS/HbAC *β* (95% CI), p	HbS/HbC/HbSC *β* (95% CI), *p*	Model F (df), *p*	*R*2
Hct (%)	30.43	−1.29 (−2.38, −0.20), 0.021	−6.40 (−10.50, −2.29), 0.002	F(2 397) = 6.95, 0.0011	0.036
MCV (fL)	77.15	−4.05 (−6.10, −2.00), < 0.001	−6.45 (−10.29, −2.61), 0.001	F(2 397) = 11.38, < 0.001	0.041
MCH (pg)	24.45	−0.89 (−1.49, −0.29), 0.004	−0.61 (−2.88, 1.67), 0.596	F(2 397) = 4.31, 0.0141	0.019
RDW‐SD (fL)	42.09	−2.80 (−4.03, –1.57), < 0.001	−1.29 (−4.43, 1.86), 0.422	F(2 397) = 10.11, 0.0001	0.05

*Note:* The “HbA mean” column represents the regression constant.

Abbreviations: *β*, unadjusted linear regression coefficient; CI, confidence interval; df, degrees of freedom (numerator, denominator); F, F‐statistic for the overall model; HbA, reference category for Hb phenotype; Hct, haematocrit; MCH, mean cell haemoglobin; MCV, mean cell volume; R2, coefficient of determination, representing the proportion of variance in the outcome explained by the model; RDW‐SD, red cell distribution width standard deviation.

**Table 4:** Multivariable linear regression of haemoglobin phenotype groups and socio‐demographic and obstetric covariates on red cell parameters.

Predictor	Category	Hct (%) *β* (95% CI), *p*	MCV (fL) *β* (95% CI), *p*	MCH (pg) *β* (95% CI), *p*	RDW‐SD (fL) *β* (95% CI), *p*
Hb phenotype	HbA (reference)	—	—	—	—
	HbAS/HbAC	−1.14 (−2.20, −0.08), 0.036	−4.05 (−6.15, −1.95), < 0.001	−.89 (−1.51, −0.28), 0.005	−2.79 (−4.03, −1.55), < 0.001
	HbS/HbC/HbSC	−6.29 (−10.66, −1.92), 0.005	−6.87 (−10.73, −3.01), 0.001	−0.69 (−3.14, 1.77), 0.583	−1.16 (−4.29, 1.96), 0.465
Age	Per year	0.05 (−0.05, 0.15), 0.313	0.06 (−0.11, 0.23), 0.487	0.05 (−0.01, 0.10), 0.095	0.01 (−0.10, 0.12), 0.860
BMI	Per kg/m^2^	0.02 (−0.08, 0.12), 0.681	0.00 (−0.20, 0.20), 0.998	−0.02 (−0.08, 0.04), 0.510	0.05 (−0.07, 0.16), 0.419
Gestational trimester	First (reference)	—	—	—	—
	Second	−1.92 (−2.86, −0.97), < 0.001	−0.03 (−1.97, 1.92), 0.980	0.03 (−0.56, 0.62), 0.920	−0.11 (−1.21, 0.99), 0.847
	Third	–3.34 (−4.88, −1.81), < 0.001	−2.74 (−5.93, 0.45), 0.092	−0.65 (−1.72, 0.41), 0.229	−2.43 (−4.38, −0.47), 0.015
Parity	Nulliparous (reference)	—	—	—	—
	Primiparous	0.67 (−0.64, 1.98), 0.313	−0.70 (−3.15, 1.76), 0.577	−0.44 (−1.23, 0.35), 0.272	−0.46 (−1.90, 0.98), 0.527
	Multiparous	−0.21 (−1.62, 1.19), 0.767	0.00 (−2.51, 2.50), 0.999	−0.33 (−1.13, 0.48), 0.427	0.35 (−1.21, 1.92), 0.657
	Grand multiparous	−1.74 (−3.81, 0.33), 0.099	−0.39 (−5.69, 4.91), 0.886	0.01 (−1.72, 1.73), 0.994	0.25 (−2.45, 2.95), 0.855
Haematinic supplementation	No (reference)	—	—	—	—
	Yes	0.24 (−0.65, 1.14), 0.594	−1.00 (−2.83, 0.84), 0.286	0.14 (−0.42, 0.70), 0.625	0.65 (−0.43, 1.72), 0.239
Model summary	F (df), *p*	F(10, 389) = 4.75, *p* < 0.001	F(10, 389) = 3.08, *p* = 0.0009	F(10, 389) = 1.82, *p* = 0.056	F(10, 389) = 3.27, *p* = 0.0005
	R2	0.106	0.052	0.037	0.075

*Note:* Robust (Huber–White) standard errors were applied to the models to address heteroscedasticity. Multicollinearity was assessed using the variance inflation factor (mean VIF = 1.30; all individual VIFs < 2.1). Reference categories for categorical covariates are shown in parentheses. HbA = reference category.

Abbreviations: *β*, adjusted linear regression coefficient; CI, confidence interval; df, degrees of freedom (numerator, denominator); F, F‐statistic for the overall model; Hct, haematocrit; MCH, mean cell haemoglobin; MCV, mean cell volume; R2, coefficient of determination, representing the proportion of variance in the outcome explained by the model; RDW‐SD, red cell distribution width standard deviation.

### 3.3. Association Between Hb Phenotype and Anaemia Among Pregnant Women

The overall prevalence of anaemia among the pregnant women in this study was high (350/400; 87.5%). Among the 350 anaemic pregnant women, the majority had moderate anaemia (231, 66.0%), followed by mild anaemia (103, 29.4%) and severe anaemia (16, 4.6%). The severity distribution differed numerically across Hb phenotype groups: severe anaemia accounted for 4.4% (11/252) of anaemic HbA phenotype group, 3.3% (3/92) of HbAS/HbAC phenotype group and 33.3% (2/6) of women in the HbS/HbC/HbSC group, although this difference did not reach statistical significance (*p* = 0.075) (Table 1).

In the univariable (unadjusted) Firth′s logistic regression analysis, no variant phenotype group was significantly associated with anaemia compared with the HbA phenotype (HbAS/HbAC: crude odds ratio [cOR] = 1.60, 95% CI: 0.76, 3.37, *p* = 0.216; HbS/HbC/HbSC: cOR = 2.14, 95% CI: 0.12, 38.64, *p* = 0.607). After adjusting for maternal age, BMI, gestational trimester, parity and haematinic supplementation in the multivariable Firth′s logistic regression model, the association between the Hb phenotype groups and anaemia remained non‐significant (HbAS/HbAC: adjusted odds ratio [aOR] = 1.61, 95% CI: 0.75, 3.47, *p* = 0.220; HbS/HbC/HbSC: aOR = 1.94, 95% CI: 0.10, 37.53, *p* = 0.660). However, gestational trimester was the only covariate independently associated with anaemia, as women in the second trimester had significantly higher odds of being anaemic (aOR = 3.45, 95% CI: 1.68, 7.09, *p* = 0.001) compared with those in the first trimester, whereas those in the third trimester showed a similar direction of effect that did not reach statistical significance (aOR = 3.07, 95% CI: 0.79, 11.84, *p* = 0.104). Maternal age, BMI, parity and haematinic supplementation were not independently associated with anaemia status (all *p* > 0.05) (Table 5).

**Table 5:** Univariable and multivariable Firth′s penalised‐likelihood logistic regression of haemoglobin phenotype groups and selected covariates on anaemia status among pregnant women.

Predictor	Category	cOR (95% CI), *p*	aOR (95% CI), *p*
Hb phenotype	HbA (reference)	1.00	1.00
	HbAS/HbAC	1.60 (0.76, 3.37), 0.216	1.61 (0.75, 3.47), 0.220
	HbS/HbC/HbSC	2.14 (0.12, 38.64), 0.607	1.94 (0.10, 37.53), 0.660
Age	Per year	0.97 (0.93, 1.02), 0.190	0.95 (0.89, 1.00), 0.067
BMI	Per kg/m^2^	0.98 (0.93, 1.04), 0.580	0.98 (0.92, 1.04), 0.527
Gestational trimester	First (reference)	1.00	1.00
	Second	3.86 (1.88, 7.90), < 0.001	3.45 (1.68, 7.09), 0.001
	Third	3.44 (0.91, 13.02), 0.069	3.07 (0.79, 11.84), 0.104
Parity	Nulliparous (reference)	1.00	1.00
	Primiparous	0.84 (0.41, 1.72), 0.634	1.07 (0.49, 2.36), 0.867
	Multiparous	1.27 (0.62, 2.62), 0.510	2.03 (0.83, 5.00), 0.122
	Grand multiparous	1.60 (0.28, 9.17), 0.598	3.15 (0.46, 21.73), 0.244
Haematinic supplementation	No (reference)	1.00	1.00
	Yes	0.68 (0.38, 1.24), 0.209	0.74 (0.40, 1.37), 0.344

*Note:*Firth′s penalised‐likelihood logistic regression was applied to the models to address complete separation in the HbS/HbC/HbSC phenotype group (which contained no nonanaemic women). Overall multivariable model: Wald *χ* ^2^(10) = 21.95, *p* = 0.015.

Abbreviations: aOR, adjusted odds ratio; CI, confidence interval; cOR, crude odds ratio; HbA, reference category; Wald *χ*2, Wald chi‐square.

## 4. Discussion

Anaemia in pregnancy remains a global health concern, particularly in sub‐Saharan Africa, and the adverse consequences for both mother and foetus may be compounded when the underlying cause is a haemoglobinopathy [41]. This study examined the association between Hb variant phenotypes and red cell parameters and assessed whether such variants were independently associated with anaemia among pregnant women after adjustment for the physiological haematological changes that accompany pregnancy. Hb variant phenotypes were associated with progressively lower red cell parameters; however, the odds of anaemia were determined primarily by gestational trimester rather than by Hb phenotype.

This study found that following the normal HbA phenotype, the most prevalent variant phenotypes were heterozygous HbAS and HbAC (25.3%). In contrast, only 1.5% of pregnant women had homozygous or compound heterozygous phenotypes HbS, HbC and HbSC. This observation aligns with reports from West Africa, where the sickle cell trait (HbAS) and HbC trait (HbAC) are relatively common because of the selective advantage against malaria [9, 42]. Similar prevalence patterns have been reported in other antenatal populations in Ghana [19, 20] and across other sub‐Saharan settings [21–23], where heterozygous Hb variant phenotypes predominate over homozygous forms. The low frequencies of HbS, HbC and HbSC observed in this study were expected, as individuals with severe haemoglobinopathies, such as homozygous HbS or compound heterozygous (HbSC) states, are less likely to reach reproductive age without clinical intervention [43–46]. In addition, although most socio‐demographic and obstetric characteristics were comparable across the Hb phenotype groups, significant differences were observed in the BMI of the pregnant women. Post hoc pairwise comparisons confirmed that the BMI of the HbS/HbC/HbSC group was significantly lower than that of both the HbA and HbAS/HbAC groups, whereas the BMI did not differ significantly between the HbA and HbAS/HbAC groups. This finding, although interpreted with caution given the small HbS/HbC/HbSC subgroup size, is consistent with a previous study involving pregnant women with sickle cell disease [47]. This has been attributed to the elevated resting energy expenditure associated with the hypermetabolic state driven by increased erythropoiesis, haemolysis, red cell turnover, chronic inflammation and cardiac demand in individuals with sickle cell disease [47, 48]. Pregnancy further amplifies metabolic demand and may widen this anthropometric gap [49]. Thus, this observation emphasises the broader nutritional and metabolic vulnerabilities that accompany homozygous and compound heterozygous Hb variants during pregnancy.

A directionally consistent inverse association between Hb phenotype groups and red cell parameters (most pronounced for Hct and MCV) was observed in this study. This finding is both biologically plausible and clinically meaningful. The heterozygous (HbAS/HbAC) group showed modest but statistically significant reductions in Hct, MCV, MCH and RDW‐SD compared with the HbA reference group, whereas pregnant women in the homozygous or compound heterozygous (HbS/HbC/HbSC) group showed substantially larger reductions in Hct and MCV. Reductions in MCH and RDW‐SD in this subgroup were in the same direction but did not reach statistical significance, most likely reflecting the limited precision afforded by the small subgroup size. Notably, RBC count, Hb concentration and MCHC did not differ significantly across the phenotype groups, suggesting that variant Hbs in this cohort primarily affected red cell volume and Hb content per cell rather than absolute red cell number or Hb concentration. These observations are consistent with the established pathophysiology of variant Hbs, in which structural abnormalities of the *β*‐globin chain (HbS, HbC) and the resulting alterations in Hb solubility, red cell membrane stability, cellular hydration and accelerated haemolysis (observed in homozygous and compound heterozygous forms) produce microcytic, hypochromic red cells [4, 50–53]. The microcytic and hypochromic profile observed in the heterozygous carriers (HbAS/HbAC) group in this study is consistent with previous reports that individuals with sickle cell and HbC traits frequently display reduced MCV and MCH despite being clinically asymptomatic [50, 54, 55]. Importantly, these associations persisted after adjusting for maternal age, BMI, gestational trimester, parity and haematinic supplementation, indicating that the Hb phenotype is independently associated with the red cell parameter profile in pregnancy, rather than reflecting confounding by socio‐demographic or obstetric factors.

The overall 87.5% prevalence of anaemia recorded in this study exceeds the World Health Organisation ≥ 40% threshold for classification as a severe public health problem, and is higher than the 51.4% rate reported among pregnant women in a Ghana Demographic and Health Survey [56, 57]. This discrepancy may reflect the facility‐based nature of our sample, regional and seasonal dietary patterns and the multifactorial aetiology of pregnancy‐related anaemia [57]. Similar Ghanaian facility‐based studies and studies elsewhere in sub‐Saharan Africa have reported anaemia prevalence exceeding 40% among pregnant women [58–61], emphasising the burden of pregnancy‐associated anaemia in this setting despite ongoing supplementation programmes.

Although Hb phenotype was independently associated with reduced red cell parameters, it was not independently associated with anaemia status when assessed using the WHO Hb cut‐off of 11.0 g/dL [35], even after adjusting for maternal age, BMI, gestational trimester, parity and haematinic supplementation. This contrasts with the findings of Cantave et al. [62]. Possible reasons for this may include the very high background prevalence of anaemia in this cohort, which likely resulted in a ceiling effect, limiting the discriminatory capacity of the binary outcome. Again, dichotomising a continuous variable, such as Hb concentration, at a fixed threshold results in a loss of information and reduces statistical power compared with analyses treating Hb as a continuous variable [63–65]. Furthermore, the very small homozygous or compound heterozygous (HbS/HbC/HbSC) subgroup substantially limited the statistical power to detect any true increase in anaemia odds, although all six pregnant women were classified as anaemic. Formally, the study was powered to detect an adjusted odds ratio of approximately 3.0 in the HbAS/HbAC group and of approximately 69 in the HbS/HbC/HbSC group; the observed estimates (aOR 1.61 and 1.94, respectively) therefore fell well below the detectable range, and these null results are more appropriately read as inconclusive than as evidence of no association. Finally, reductions in Hb concentrations of heterozygous Hb variant carriers, such as HbAS/HbAC, are often modest and may not consistently cross the diagnostic anaemia threshold without another underlying factor [55, 66]. Nevertheless, when the 350 anaemic women were classified by WHO severity, the proportion classified as severe was disproportionately higher in the HbS/HbC/HbSC phenotype group than in the HbAS/HbAC or HbA groups. Although this difference was not statistically significant, the directional pattern aligns biologically with the chronic haemolytic anaemia characteristic of homozygous and compound heterozygous haemoglobinopathies [44, 51]. This observation underscores the potential for clinically meaningful effects in this small subgroup, despite the statistical underpowering.

Gestational trimester emerged as the only variable consistently and independently associated with both reduced red cell parameters and higher odds of anaemia. Specifically, Hct was 1.92% lower in the second trimester and 3.34% lower in the third trimester than in the first trimester, and the odds of anaemia in the second trimester were more than three‐fold higher than those in the first trimester. These findings are well supported in the literature and reflect the physiological haemodilution of pregnancy, where plasma volume expansion exceeds the red cell mass increase and produces a relative reduction in Hb concentration and Hct that is most evident in the second and early third trimesters [67, 68]. This physiological haemodilution due to gestational age may also have contributed to the misclassification of true iron‐replete women as anaemic when a single Hb cut‐off is applied across trimesters, and supports the use of trimester‐specific reference thresholds in ANC [69–71]. Antenatal Hb variant screening is already a component of standard ANC in Ghana, and the present data are not a test of that policy. However, it is not established whether phenotype‐aware interpretation of red cell parameters improves maternal outcomes; this question requires evaluation of diagnostic yield, downstream clinical benefits and cost‐effectiveness in larger multicentre studies.

### 4.1. Limitations

This study had some limitations to note. Although participants were screened for malaria parasitaemia by blood‐film microscopy (which may not detect submicroscopic infections) and intestinal parasites at enrolment, baseline iron status was not assessed, and iron biomarkers, folate, vitamin B12 and inflammatory markers were not measured; hence, residual confounding by these factors cannot be excluded, particularly in interpreting the high overall anaemia prevalence. HPLC was not performed to rule out thalassaemia traits, and peripheral blood film for morphological assessment was not performed. In addition, cellulose acetate electrophoresis at pH 8.4 does not reliably distinguish HbC from HbE and HbA₂ owing to their comigration on this medium; HPLC or capillary electrophoresis would be required for definitive confirmation of HbC. Gestational age was taken from the ANC records rather than rederived for the study; therefore, the dating method applied to each participant could not be verified. Haematinic supplementation was self‐reported at a single point of antenatal contact and is therefore subject to recall bias, with no information recorded on drug name, formulation, dose, duration or adherence. Antibiotic use in the 30 days preceding sampling was also not systematically documented, although all six pregnant women with homozygous or compound heterozygous phenotype were confirmed to have been free of painful crisis over that period. Notably, only one of these six pregnant women reported current haematinic use, since folic acid supplementation is recommended in sickle cell disease, this may reflect the timing of the interview relative to the initiation of routine antenatal supplementation rather than true nonprovision, but it cannot be verified from the present data. Furthermore, the small number of participants with homozygous or compound heterozygous phenotypes produced wide CIs around several of the estimates, particularly for MCH, RDW‐SD and the anaemia outcome. The minimum detectable effects for this subgroup are reported under statistical power and precision, and the absence of statistically significant reductions in MCH and RDW‐SD cannot be taken as evidence that none exists. Finally, the single‐facility, hospital‐based nature of the study may limit its generalisability to other clinical settings or community‐based pregnant populations in Ghana.

## 5. Conclusion

This study found that Hb variant phenotypes are independently associated with reduced red cell parameters such as Hct, MCV, MCH and RDW‐SD among pregnant women, after adjustment for maternal and obstetric covariates. The reductions were numerically larger in homozygous and compound heterozygous phenotypes, although the limited subgroup size precluded statistically significant findings for some indices. Despite these associations with reduced red cell parameters, Hb phenotype was not independently associated with anaemia status defined by the WHO Hb cut‐off, and gestational trimester emerged as the dominant correlate of anaemia in this cohort. These findings highlight the need for phenotype‐ and trimester‐aware interpretation of red cell parameters and anaemia in pregnant women carrying variant Hb phenotypes. Whether such an interpretation improves maternal outcomes was not tested here and should be evaluated in larger multicentre studies. Future studies with larger samples of homozygous and compound heterozygous carriers, HPLC screening for thalassaemia traits and assessment of iron status biomarkers are needed to clarify the relative contributions of inherited and acquired factors to anaemia in this population.

## Author Contributions

All the authors involved in this study played a significant role in various aspects, such as conception, methodology, participant recruitment and sample collection, laboratory investigations, statistical analysis and drafting of this manuscript. Furthermore, they provided final approval for the version to be published, agreed to the journal to which the article was submitted and are accountable for all aspects of this work. Conceptualisation: Gabriel Abbam, Bertrand Nii Martey Nmeterson. Methodology and design: Charles Nkansah, Samuel Kwasi Appiah, Samira Daud. Participant recruitment and sample collection: Bertrand Nii Martey Nmeterson, Sophia Owusua Amankwah. Laboratory investigations: Gabriel Abbam, Bertrand Nii Martey Nmeterson, Sophia Owusua Amankwah, Muniru Mohammed Tanko. Statistical analysis: Gabriel Abbam, Moses Banyeh. Resources: Gabriel Abbam, Bertrand Nii Martey Nmeterson, Sophia Owusua Amankwah. Supervision: Gabriel Abbam, Simon Bannison Bani. Drafting of original manuscript: Gabriel Abbam, Bertrand Nii Martey Nmeterson. Writing—review and editing: Ejike Felix Chukwurah, Samira Daud, Charles Nkansah, Felix Osei‐Boakye, Samuel Kwasi Appiah, Muniru Mohammed Tanko, Simon Bannison Bani.

## Funding

No funding was received for this manuscript.

## Conflicts of Interest

The authors declare no conflicts of interest.

## Supporting information


**Supporting Information** Additional supporting information can be found online in the Supporting Information section. Table S1: Distribution of stool, malaria parasite and urinalysis findings among pregnant women (*N* = 400). Table S2: Comparison of body mass index by Hb phenotype groups. Table S3: Comparison of red cell indices by haemoglobin phenotype with significant pairs.

## Data Availability

The raw data supporting the findings of this study are available in Figshare with the identifier 10.6084/m9.figshare.32420322.
